# Completeness and overlap in open access systems: Search engines, aggregate institutional repositories and physics-related open sources

**DOI:** 10.1371/journal.pone.0189751

**Published:** 2017-12-21

**Authors:** Ming-yueh Tsay, Tai-luan Wu, Ling-li Tseng

**Affiliations:** Information and Archival Studies, National Cheng-Chi University, Taipei, Taiwan; International Nutrition Inc, UNITED STATES

## Abstract

This study examines the completeness and overlap of coverage in physics of six open access scholarly communication systems, including two search engines (Google Scholar and Microsoft Academic), two aggregate institutional repositories (OAIster and OpenDOAR), and two physics-related open sources (arXiv.org and Astrophysics Data System). The 2001–2013 Nobel Laureates in Physics served as the sample. Bibliographic records of their publications were retrieved and downloaded from each system, and a computer program was developed to perform the analytical tasks of sorting, comparison, elimination, aggregation and statistical calculations. Quantitative analyses and cross-referencing were performed to determine the completeness and overlap of the system coverage of the six open access systems. The results may enable scholars to select an appropriate open access system as an efficient scholarly communication channel, and academic institutions may build institutional repositories or independently create citation index systems in the future. Suggestions on indicators and tools for academic assessment are presented based on the comprehensiveness assessment of each system.

## Introduction

Assessing research performance, including research productivity and citation-based research impact, has become an important issue for scholars and research institutions. The rapid development of the Internet has facilitated the diversification of scholarly communication channels, which has not only altered the scholarly communication environment but also further promoted the open access movement. Academic literature is abundant and has been rapidly circulating in the cyber world. This has led to the expansion and reform of traditional bibliometric research and the consequent development of webometrics. The research data analyzed by webometrics come from the cyber world; therefore, websites, search engines and other open access systems can all be webometric research subjects or research tools. How webometrics precisely and rigorously analyze and evaluate scholars, institutions and academic literature has been a major research topic. As the research foundation of webometrics, open access systems differ from one another with respect to their objectives of construction, functions, and scope of collection. Therefore, in webometric studies, it is necessary to understand the differences among open access systems if they are to be used as research tools. This is the objective of this study.

The “open access” environment has been collectively developed by academic communities in recent years. Open access systems (including institutional repositories) constructed by scholars and non-profit organizations, such as academic institutions and libraries, may also possess the function of citation indexes. For example, in the computer science domain, CiteSeer and Citebase constructed a citation index for their free online academic publications, including arXiv.org, which has archived research literature in such domains as physics, mathematics, computer science, and biostatistics [1]. Furthermore, comparing with commercial databases and open access systems, search engines are more important tools for many users in information search. How should users choose among search engines and open access systems? What are the differences among these sources? What are the pros and cons in terms of the completeness and overlap of the collected data?

Based on the above-described background and motivations, this study compares the completeness and overlap of the data collected in open access systems using webometric methods. The open access systems selected in this study include two search engines, i.e., Google Scholar and Microsoft Academic; two full-text institutional repositories, i.e., OpenDOAR and OAIster; and two physics-related repositories, i.e., arXiv.org and Astrophysics Data System. The following objectives are expected to be achieved in this study.

The academic literature published by the 2001–2013 Nobel Laureates in Physics is used as the research sample for comparing the completeness and overlap of the data collected in various open access systems.

Based on the results of the aforementioned comparison, this study analyzes the pros and cons of the above-mentioned open access systems and makes recommendations according to the analysis results to serve as a guide for scholars and researchers of webometrics for selecting information systems.

Poyer- defines the overlap of journal articles as the same journal article being collected and indexed or abstracted in two or more databases. Overlap also refers to the rate at which the same article is collected in two or more databases simultaneously; this is the definition of overlap used in this study [2].

Hood- conducted an overlap study of all databases under the Dialog system. He used the theme of fuzzy set theory, downloading literature from all databases in the Dialog system that included “fuzzy” data and selecting the literature related to “fuzzy set theory.” Altogether, he chose more than 30,000 data items between 1965 and 1993 from about 100 different databases. Hood removed the errors, unified all the fields and ultimately selected 15,644 items. He then conducted a comparative study of the overlap distribution, records with the most overlaps, sole records, duplicate records, and the overlap among the top ten databases. He found considerable differences in the overlap records among the databases; of the 15,644 data items, 9,897 (63.26%) were covered in only one database, 1,922 data items (12.29%) were covered in two databases, and 5 data items (0.03%) were covered in 12 databases. In addition, Hood found internal duplicates in 28 databases, with MATHSCI having the most (239 items). The reasons for the high rate of internal duplicates were that both original articles and original papers with abstracts were covered in the database, and the same article might be collected by multiple journals. Hood finally explained the relative overlap among the various databases, noting that the relative overlap of INSPEC and SCISEARCH exceeded 40%, which was the highest of all the databases [3].

Rather, Lone and Shah- conducted an overlap study on five search engines (i.e., Google, AltaVista, HotBot, Scopus and Bioweb) using the retrieval results of 20 subjects in biotechnology. The findings indicated that HotBot, followed by Google, had the highest degree of overlap with the other search engines. The authors also found that compound and complex retrieval produced more overlapping results [4]. Esmaeil, Kiaie and Ketab compared the overlap of six commonly used search engines suggested by Searchenginewatch.com. They found that among the different search engines, the overlap rate of Yahoo with the other engines was the highest, about 40%. The recall rate of the physics journals of Curry Guide was 77.1%, and its overlap rate with the other five systems was 43.7%. Taking the physics domain as an example, the data in Meta Search Engine were the most complete among the six open access search engines [5].

Wang, et al conducted an analysis and comparison of four search engines—Google, Yahoo, Bing and Ask.com—using breast cancer as the keyword in their search. The retrieval results of the four big search engines with respect to the six standards of breast cancer all ranked in the first 30. A pairwise comparison revealed an overlap rate of over 50% in the data collected in these engines; in terms of the satisfaction of users, each of the four search engines emphasized different types of content. Bing had the highest user satisfaction rate, followed by Yahoo and Google, with Ask.com ranked last. The authors concluded that searching with the appropriate search engines according to information needs increases efficiency [6].

The research on completeness and overlap has become extensive; it focuses on data providers (such as publishers, databases and search engines) as well as various types of data sources (such as journals, patents, and internet resources). The completeness and overlap rate of data are also important factors for users to select an appropriate information-searching system.

## Research methods and limitations

### Research methods

This study collected papers published by the Nobel Laureates in Physics, 2001–2013, and then produced a publication list to serve as the research sample for a comparative study of the six aforementioned open access systems (two search engines, Google Scholar and Microsoft Academic; two full-text institutional repositories, OpenDOAR and OAIster; and two physics-related open access systems, arXiv.org and Astrophysics Data System).

The open access movement emerged in the 1990s after the Internet began to flourish. The systems used in this study were constructed in the following years: Google Scholar, 2004; Microsoft Academic, 2016; OpenDOAR, 2005; OAIster, 2002; arXiv.org, 1991; and Astrophysics Data System, 1992. The differences in the years of construction might lead to differences in the quantity of the data collected in each system; systems constructed earlier might cover data published in earlier years than those constructed later.

To create the research sample of this study, i.e., the publication list of the Nobel Laureates in Physics, 2001–2013, it was necessary to retrieve the personal publications disclosed by these renowned scholars in physics. Moreover, an author search was performed to ensure the publication list of the research sample being complete and accurate. The Nobel Laureates’ publication lists were cross-referenced with their publication data obtained from various Internet resources, including the websites of their won or their research teams, and the websites of the institutions with which they are affiliated.

The names of the Nobel Laureates were searched separately in Google Scholar, Microsoft Academic, OAIster, arXiv.org, and Astrophysics Data System. OpenDOAR was not searched in this step as it does not have a search function. After authors with identical names (not the scholars intended for this research) were identified and eliminated, the retrieval results from the systems were exported and collated. The following fields were exported and analyzed to identify the correctness of the data: bibliographic records, title, author, publication year, publisher, journal title, volume and issue, and number of pages.

To avoid errors in the bibliographic data that arise during massive downloads, and to collect complete bibliographic data, the bibliographies were downloaded according to the complete descriptive format. The data processed by bibliometric methods mostly include text and numeric data. As different systems have different descriptive formats, incomplete descriptions and different description formats might arise when downloading bibliographic data. Therefore, the data must first be rectified through a manual search to enable the original massive and jumbled bibliographic data to be standardized and the overlapping bibliographies retrieved from different databases to be removed through an Excel spreadsheet.

After being collected and processed, the bibliographic data were manually verified and compared to ensure their accuracy, as there might be errors such as typographical errors in bibliographies, the inclusion of research papers unrelated to physics, the author’s use of an alias, and different titles and descriptive formats for the same journal. The bibliographic list created after the overlaps were eliminated and the bibliographic data were processed served as the publication list of the Nobel Laureates collated with all of the databases.

The bibliographic titles of the Nobel Laureates were searched in the six open access systems studied. The search results for each title on each system were then individually recorded, including the descriptive errors and bibliography overlaps, which served as the basis for evaluating the completeness and overlap of the bibliography of the Nobel Laureates. Finally, the results and reviews of the six systems were organized, statistically assessed, and compared.

### Limitations

This study was based on the journal papers published by the Nobel Laurates in Physics from 2001–2013. These scholars may be considered as leaders in physics research and the content of these papers may be more innovative and quite different from vast majority of papers published by non-Nobel Laurates. The findings reported in this study may be limited to the papers published by the Nobel Laurates in Physics from 2001–2013.

## Research results

### Completeness of six open access systems

**Fig 1:**
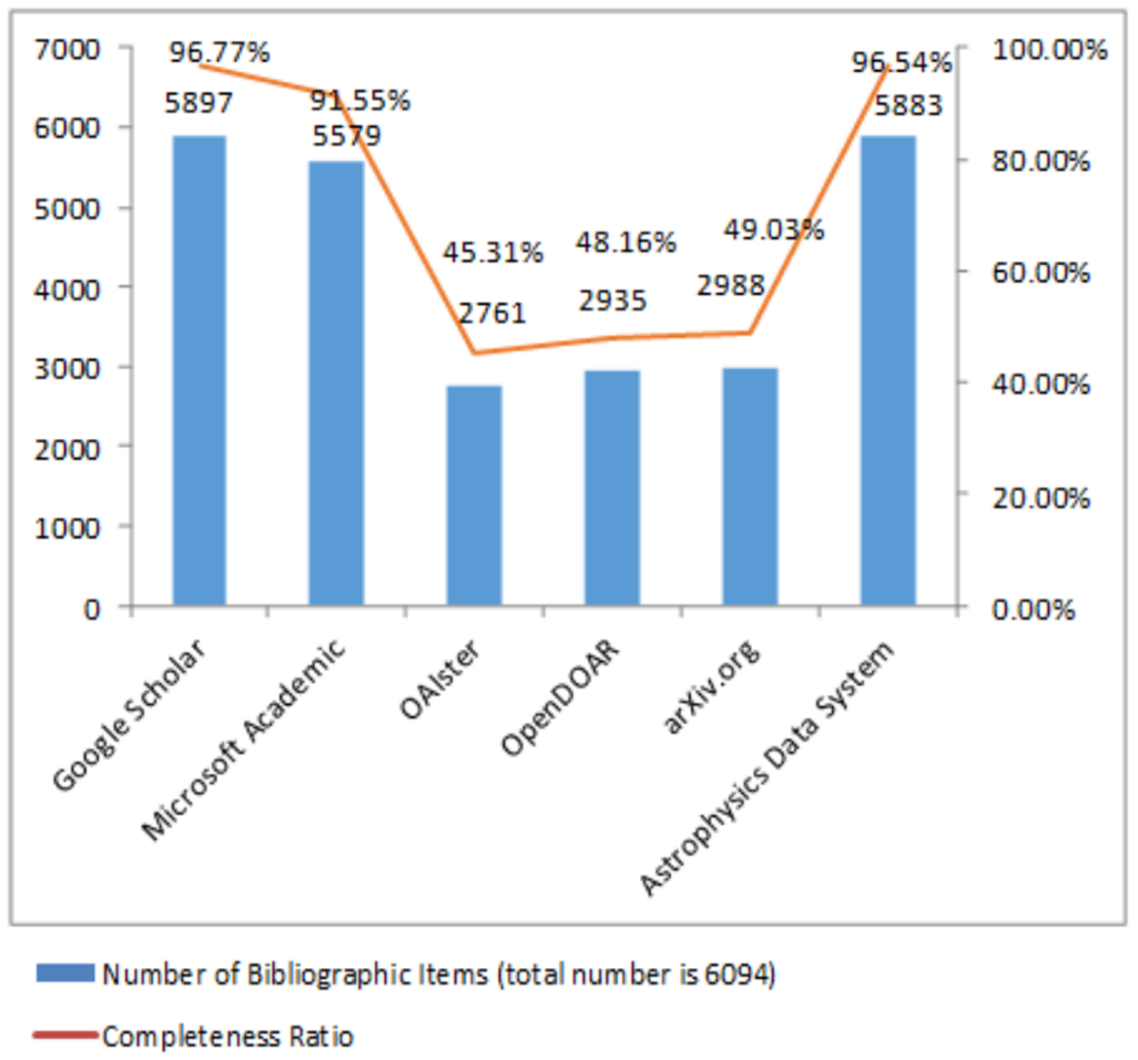
Completeness of the sample publication list in physics in the open access systems.

Based on the publication list of journal papers published by the Nobel Laureates in physics, 2001–2013, as shown in the S1 Appendix, a search of a total of 6094 items was performed in the open access systems. The amount and completeness of the bibliographic data are shown in Fig 1. Google Scholar has the most complete results, with 5897 bibliographic data items, covering 96.8% of the publication list. Astrophysics Data System is almost on par with Google Scholar, covering 96.5% of the publication list. Microsoft Academic includes 91.6% of the data, and OAIster ranks last. In the open access systems, the disciplinary databases Astrophysics Data System and arXiv.org include the most relevant items (49% of the publication list items), although the completeness of their collections differ immensely. The arXiv.org collection is not up to par with the search engines, with a difference in coverage of about 35%-47%; however, the completeness of Astrophysics Data System is very close to that of Google Scholar. The completeness of the institutional repositories is not as high as arXiv.org, and OpenDOAR, with a completeness of 48%, is more complete than OAIster, at 45%.

The reasons for Google Scholar having the most complete data is that it indexes the literature in the network world; its sources of literature include academic publishers, the institutional repositories of all universities and open access systems. Moreover, Google Scholar has indexed the open access system of Astrophysics Data System, rendering its bibliography the most complete.

The completeness of the databases and the systematically collected data with regard to the physics literature rank as follows, starting with the highest: Google Scholar, Astrophysics Data System, Microsoft Academic, arXiv.org, OpenDOAR, and OAIster.

### Overlap of six open access systems

There are two types of system collection overlap: internal and external. Internal overlap, or self-overlap, occurs when duplicated bibliographies exist within each system. External overlap, or relative overlap, occurs when the same bibliographic items are covered in two or more open access systems.

### Internal overlap

Table 1 shows the internal overlap of the bibliographic data from the retrieval of the list of works by the Nobel Laureates from the six open access systems. arXiv.org and the Astrophysics Data System, which are in the physics discipline, have lower internal overlap percentages than other open access systems. arXiv.org has the least internal overlap, i.e., no internal overlap (0%), and Astrophysics Data System has an internal overlap rate of only 1.3%. Google Scholar and OpenDOAR have the highest internal overlap rates, at 92.7% and 46.2%, respectively. Regarding the internal overlap rate of the institutional repository systems, OpenDOAR has a rate of 46.2% and OAIster 13.8%.

**Table 1:** Internal overlap of the publication list of the 2001–2013 Nobel Laureates in Physics in the open access systems.

	Internal overlap			
Open access system	Bibliographic items	Overlap Items	Overlap Percentage (%)	Completeness percentage/ Overlap percentage
Google Scholar	5897	5464	92.66	1.044
Microsoft Academic	5579	662	11.87	7.713
OAIster	2761	381	13.80	3.283
OpenDOAR	2935	1355	46.17	1.043
arXiv.org	2988	0	0	0
Astrophysics Data System	5883	79	1.34	72.05

A Pearson correlation test between the bibliographic items and internal overlap items results in a correlation coefficient of 0.418 with p>0.05. This suggests that there is an intermediate relationship between the bibliographic items and internal overlap items but insignificant.

The reason for the high degree of internal overlap of Google Scholar is that it indexes online literature from across the world. In Google Scholar, for the publication list of the Nobel Laureates, only 433 documents contained only one source, while up to 92.7% contained two or more bibliographic sources. OpenDOAR uses the search functionality provided by Google Custom Search for repository content retrieval and searches the contents of all open access repositories. The retrieval results may include several bibliographic sources. Since the internal overlap mentioned above is due to multiple bibliographic sources, the situation in each system may differ. The internal overlap rate of Microsoft Academic is 11.87%. Evidently, internal overlap records are caused by title input discrepancies (e.g., a missing article in the title, special symbols recorded in spelling) or the lack of bibliographic information (e.g., publication title, volume, pages). The difficulty of distinguishing one entry from another has prevented the system from deleting duplicate bibliographic records.

It is also interesting to examine the ratio of completeness percentage in Fig 1 to the internal overlap percentage in Table 1. The last column of Table 1 lists this ratio. The Table shows three groups. Google Scholar and OpenDOAR are in the same group with the ratio about 1, Microsoft Academic and OAIster are the intermediate group with a ratio of 3 to 8, and Astrophysics Data System presents the largest ratio of 72.05. Astrophysics Data System demonstrates a merit with high completeness percentage and relatively low internal overlap percentage.

### External overlap

Table 2 demonstrates that Google Scholar is very similar to Microsoft Academic, with an overlap rate of 92.4%, and it has the highest overlap rate with Astrophysics Data System at 99.76% (5883 overlapping bibliographies). Its overlap rates with arXiv.org, OAIster and OpenDOAR are 50.67%, 46.82% and 49.77%, respectively.

**Table 2:** Percentage of external overlap of the publication list of the 2001–2013 Nobel Laureates in Physics in the open access systems (cross-column system and cross-reference system).

	Google Scholar	Microsoft Academic	arXiv.org	OAIster	OpenDOAR	Astrophysics Data System
Google Scholar	--	92.35	50.67	46.82	49.77	99.76
Microsoft Academic	97.62	--	48.66	46.21	48.09	97.08
arXiv.org	100	90.86	--	87.08	97.76	100
OAIster	100	93.37	94.24	--	98.08	100
OpenDOAR	100	91.41	99.52	92.27	--	100
Astrophysics Data System	100	92.06	50.79	46.93	49.89	--

Microsoft Academic has high bibliographic overlap rates with Google Scholar (97.08%) and Astrophysics Data System (97.08%). With regard to other open access systems, arXiv.org, OAIster, and OpenDOAR, Microsoft Academic has the most overlap with arXiv.org. For the other open access systems, the bibliographic overlap rate is approximately 46%-49%, without a significant difference between the open access systems.

OAIster has an overlap rate of 100% with Google Scholar and Astrophysics Data System, which is very similar to the overlap rate of arXiv.org with these two systems.

Similarly, OpenDOAR has a 100% overlap rate with Google Scholar and Astrophysics Data System. Regarding the overlap rates of the other open access systems, the following results were obtained: the overlap rates of arXiv.org and OAIster with OpenDOAR are 87.08% and 97.76%, respectively; those of OAIster and arXiv.org with OpenDOAR are 94.24% and 98.08%, respectively; and those of OpenDOAR and arXiv.org with OAIster are 99.52% and 92.27%, respectively. While the overlap rate between arXiv.org and OAIster is only 87.08%, the overlap rate with other systems is over 90%. This indicates the overlap rate of the open access systems with another similar source is extremely high.

arXiv.org overlaps with OAIster at a rate of 87.08%, and it overlaps with other open access systems at higher rates (all over 90%, except OAIster). The external overlap rate of arXiv.org with Google Scholar and Astrophysics Data System is 100%. Astrophysics Data System had a high overlap with Google Scholar (100%) and Microsoft Academic (92.06%). The bibliographic overlap rate between Astrophysics Data System and other open access systems varies from 47% to 51%, a lower overlap rate compared with other open access systems (e.g. arXiv.org, OAIster and OpenDOAR).

In summary, arXiv.org, OAIster, OpenDOAR and Astrophysics Data System all overlap with Google Scholar at a rate of 100%, suggesting that the information found in other systems can almost always be obtained from Google Scholar. Additionally, Microsoft Academic contains more than 90% of the information from other systems (with an overlap rate exceeding 90%). arXiv.org, OAIster and OpenDOAR overlap with Astrophysics Data System and Google Scholar at a rate of 100%, they overlap with Microsoft Academic at rates of approximately 91%-93%.

The reason for the high bibliographic overlap rate of Google Scholar and Astrophysics Data System with other databases and systems is that Google Scholar has the most extensive bibliography and abundant information data resources. During the research process, it was noted that most resources from Google Scholar come from Astrophysics Data System (which has an overlap rate with Google Scholar of 100%). Astrophysics Data System includes information from databases such as Astronomy and Astrophysics, Physics, and arXiv.org, which is likely the reason behind the 100% overlap rate of arXiv.org, OAIster, and OpenDOAR with Google Scholar.

arXiv.org and OAIster overlap with OpenDOAR at rates of 97.8% and 98.1%, respectively. OpenDOAR’s bibliographic sources include Europe PubMed Central, CERN Document Server and arXiv.org, while OAIster’s bibliographic sources are mostly from academic institutions, which may result in bibliographic duplication/overlap.

## Conclusion and suggestions

In terms of the extensiveness and integrity of data in relation to the physics literature, the ranking is as follows: Google Scholar, Astrophysics Data System, Microsoft Academic, arXiv.org, OpenDOAR and OAIster. Google Scholar has the most complete data coverage (96.8%), closely followed by Astrophysics Data System (96.5%); Microsoft Academic’s data coverage is slightly lower (91.6%), while OAIster’s integrity is the lowest (45.3%). Among the physics open access systems, Astrophysics Data System and arXiv.org have the highest level of data coverage (49%), although the systems differ from one another in terms of the information included. The institutional repository system coverage is not as good as arXiv.org’s, and among them, OpenDOAR’s has a coverage rate of 48%, which is better than that of OAIster’s rate of 45%.

arXiv.org has the lowest internal overlap rate (0%). Meanwhile, arXiv.org and Astrophysics Data System, both physics-related systems, have less internal overlap than other open access systems. In addition, Astrophysics Data System demonstrates a merit with high completeness percentage and relatively low internal overlap percentage. Google Scholar and OpenDOAR have the most internal overlap, largely due to their various sources of bibliographic data; as a result, the bibliographies under each system also differ (one journal from different bibliographic sources would show different index information). Since Google Scholar indexes the international literature online, 92.7% of entries contain two or more bibliographic sources. OpenDOAR uses the search function provided by Google Custom Search to search every open access repository, which might also cause several bibliographic sources all leading to one journal.

The external overlap rate of arXiv.org, OAIster, and OpenDOAR with Google Scholar and Astrophysics Data System is 100%, identical to the overlap of Astrophysics Data System and Google Scholar. Over the course of the study, it was noted that the main bibliographic data in physics of Google Scholar are from Astrophysics Data System, including Astronomy and Astrophysics, Physics and arXiv.org. This might have resulted in the 100% collection overlap of the data in arXiv.org, OAIster and OpenDOAR with the data in Google Scholar and Astrophysics Data System.

Additionally, more than 90% of the data in other systems can be found in the Microsoft Academic search engine. Therefore, for physics literature, a user is able to retrieve extensive data using a search engine such as Google Scholar or Microsoft Academic, as well as Astrophysics Data System. While all three open access systems have the full-text link function, Google Scholar and Microsoft Academic provide users with links to the full text of the journal (PDF files and web pages) if found. In addition to the scanned early physics literature for the full-text search, Astrophysics Data System also allows full access to open access journals and links to arXiv.org preprinted electronic documents. If these three open access systems do not provide the full text of the literature, the user may purchase them from commercial databases.

It should be noted the above conclusions are drawn based on the journal articles published by the Nobel Laureates in Physics, 2001–2013. The same conclusion may not be applicable to other subjects due to the accessibility of topics in variety of search engines. As the construction year of the six open access systems are different, the completeness of search results may also be different.

Based on the findings of this study, the following suggestions may be provided for the open access systems studied in the present work.

The open access systems should enhance their search functions and provide unique retrieval mechanisms or value-added content. Astrophysics Data System provides quotation search, author networks, documents and research concepts with graphics (which should be adopted by other open access systems) and is also worthy of other open access systems. Open access systems should also implement bibliographic quality control and use a variety of screening mechanisms to eliminate unsuitable or incomplete bibliographic information to maintain the system data quality. The open access system should also overcome the problems of invalid or incorrect links to avoid irrelevant search results or search results not linked to the source site.

Since modern computer technology and large-data processing technology are already mature, future research on webometrics and collection overlap, if conducted with research methods such as data mining and/or text mining, will be beneficial to quantitative research.

## Supporting information

S1 Appendix Nobel Laureates in Physics (2001–2013). (DOCX)
