# Supplementary material for: Completeness and overlap in open access systems: Search engines, aggregate institutional repositories and physics-related open sources
Source: PLoS One. 2017 Dec 21;12(12):e0189751. doi: 10.1371/journal.pone.0189751 (PMC5739406; doi:10.1371/journal.pone.0189751)
Supplement: S1 Appendix — (DOCX) [file pone.0189751.s001.docx]

S1 Appendix Nobel Laureates in Physics (2001 – 2013)

| Year | Names of Nobel Laureates |
| --- | --- |
| 2013 | François Englert and Peter W. Higgs |
| 2012 | Serge Haroche and David J. Wineland |
| 2011 | Saul Perlmutter, Brian P. Schmidt and Adam G. Riess |
| 2010 | Andre K. Geim and Konstantin Novoselov |
| 2009 | Charles Kuen Kao, Willard S. Boyle and George E. Smith |
| 2008 | Yoichiro Nambu, Makoto Kobayashi and Toshihide Maskawa |
| 2007 | Albert Fert and Peter Grünberg |
| 2006 | John C. Mather and George F. Smoot |
| 2005 | Roy J. Glauber, John L. Hall and Theodor W. Hänsch |
| 2004 | David J. Gross, H. David Politzer and Frank Wilczek |
| 2003 | Alexei A. Abrikosov, Vitaly L. Ginzburg and Anthony J. Leggett |
| 2002 | Raymond Davis Jr., Masatoshi Koshiba and Riccardo Giacconi |
| 2001 | Eric A. Cornell, Wolfgang Ketterle and Carl E. Wieman |

Source：All Nobel Prizes in Physics. Nov. 24, 2013. Retrieved form: <http://www.nobelprize.org/nobel_prizes/physics/laureates/index.html>
